# Social, spatial, and temporal organization in a complex insect society

**DOI:** 10.1038/srep13393

**Published:** 2015-08-24

**Authors:** Lauren E. Quevillon, Ephraim M. Hanks, Shweta Bansal, David P. Hughes

**Affiliations:** 1 Center for Infectious Disease Dynamics, Penn State University, University Park, Pennsylvania, USA; 2 Department of Biology, Penn State University, University Park, Pennsylvania, USA; 3 Department of Statistics, Penn State University, University Park, Pennsylvania, USA; 4 Department of Biology, Georgetown University, Washington, D.C., USA; 5 Fogarty International Center, National Institutes of Health, Bethesda, MD, USA; 6 Department of Entomology, Penn State University, University Park, Pennsylvania, USA

## Abstract

High-density living is often associated with high disease risk due to density-dependent epidemic spread. Despite being paragons of high-density living, the social insects have largely decoupled the association with density-dependent epidemics. It is hypothesized that this is accomplished through prophylactic and inducible defenses termed ‘collective immunity’. Here we characterise segregation of carpenter ants that would be most likely to encounter infectious agents (i.e. foragers) using integrated social, spatial, and temporal analyses. Importantly, we do this in the absence of disease to establish baseline colony organization. Behavioural and social network analyses show that active foragers engage in more trophallaxis interactions than their nest worker and queen counterparts and occupy greater area within the nest. When the temporal ordering of social interactions is taken into account, active foragers and inactive foragers are not observed to interact with the queen in ways that could lead to the meaningful transfer of disease. Furthermore, theoretical resource spread analyses show that such temporal segregation does not appear to impact the colony-wide flow of food. This study provides an understanding of a complex society’s organization in the absence of disease that will serve as a null model for future studies in which disease is explicitly introduced.

Social insects are paragons of self-organized complex systems^1^,^2^,^3^,^4^,^5^. Individuals interact to produce sophisticated colony-level behaviour that is more than, and not necessarily predictable from, the behaviour of the individuals that create it^3^. This emergent behaviour, such as honeybees “democratically” choosing between nest sites^6^ or ants creating elaborate living architectures in response to environmental obstacles^7^, has likely contributed to the ecological success of the social insects as a whole. Therefore, it remains imperative to understand how behaviours at the scale of the individual and at the scale of the colony dynamically influence each other, especially given the importance of functional roles in social insect colonies^8^. Such understanding is salient in the face of perturbation, where changes at the individual level due to disease or predation may have cascading consequences for the entire colony.

Disease is an especially relevant perturbation for social insects because it has been suggested that a significant cost of high-density living is increased disease risk^9^,^10^,^11^,^12^,^13^,^14^. Though social insect colonies have both higher density and a higher average genetic relatedness than other animal groups^15^, their ecological dominance over significant evolutionary time^15^ suggests that they appear to have effectively adapted to mitigate the presumed negative cost of disease. This is not because they lack infectious agents- social insects are host to a wide array of pathogens and parasites^10^,^13^,^16^,^17^ (Table S1) that have several means of gaining entrance to and spreading within the colony. Rather, social insects are thought to mitigate intense infection pressures through a series of standing and inducible defenses termed ‘social’ or ‘collective’ immunity^18^,^19^,^20^. These defenses range from the immunological to the behavioural, including how colonies are spatially organized and which tasks are allocated to different workers^20^,^21^,^22^,^23^.

The social and spatial segregation of workers most susceptible to encountering infectious agents is often cited as a mechanism of disease prophylaxis in social insect colonies^20^,^24^,^25^. However, many of these workers (i.e. foragers) are also responsible for the delivery of beneficial substances, such as food and antimicrobial compounds (e.g. tree resin)^26^ into the colony and such segregation could impact the flow of beneficial resources^27^. Indeed, even seemingly harmful interactions, such as engaging in trophallaxis or allogrooming with a nest mate that has been exposed, can lead to the transfer of either potential immune elicitors^28^ (passive immunity) or low doses of inoculate that can lead to the mounting of a protective immune response^29^ (active immunity). This is complicated further by the fact that the cost-benefit ratio of interacting with an exposed nest mate likely depends on the host-pathogen system involved^30^. Thus, understanding how colonies have balanced the opposing demands of maximizing the spread of beneficial resources while minimizing the transmission of pathogens leading to disease remains an important aim in studies of both social insects and social organisms as a whole.

A first step to understanding this balance in social insects is to determine if the social and spatial segregation of foragers does indeed occur in the absence of disease. Empirical work done in the past two decades have investigated various aspects of social insect colony organization through social and spatial lenses (Table S2). Many studies have used proximity networks^31^,^32^,^33^ to understand worker spatial segregation. Exciting technological advances have revolutionized the resolution with which we can measure social insect spatial segregation^34^,^35^ and these studies have also confirmed the relative segregation of workers performing tasks outside the colony from those remaining within. Network studies based on social interactions rather than just spatial proximity have been harder to come by, as observing individual behaviour within a realistic colony setting remains a formidable task. Of those studies that have explicitly measured social interactions, antennation networks have been used to investigate how colony organization impacts information flow^8^,^36^ and trophallaxis networks have also revealed evidence for ‘organizational immunity’ in colony food flow patterns^37^,^38^,^39^. Most recently, analytical advances have allowed for the inclusion of temporal information in such social networks^27^,^36^. Understanding the timing of interactions is crucial for accurately understanding how food, information, and disease dynamically flow through social insect colonies.

Thus, while we have been acquiring knowledge of colony organization across many different social insect systems, we haven’t yet integrated this work across social, spatial, and temporal scales in a single study system. What would be useful now is a system in which such integration exists that can be manipulated through experimental infection in subsequent work. To that end, here we characterise the basis for standing organizational immunity through forager segregation in colonies of the black carpenter ant, *Camponotus pennsylvanicus,* using a suite of social, spatial, and temporal analyses. The ant *C. pennsylvanicus* is widespread in the northeastern USA and has evolved to nest inside dead trees^40^. We mimic this by maintaining colonies inside wood under complete darkness (video S1).

We first classify ants into functional categories based on whether they are performing or have previously performed tasks outside the nest (which translates to elevated disease risk). Next, we look at the oral exchange of food (trophallaxis) as the key social interaction of interest because colonies must balance efficient resource flow (food, antimicrobial compounds, information) with mitigating disease spread^36^. If social segregation does occur, we would expect to see its signature represented in the trophallaxis interactions between ants that have been outside and those that have remained buffered within the relatively protected confines of the colony^18^. To facilitate comparison of how trophallaxis between ant functional groups could impact potential disease risk, we borrow the concept of ‘person-time’ used in calculating epidemiological incidence rates^41^. Next, we incorporate individual movement data to assess whether spatial segregation is present in the absence of disease. Finally, we incorporate the time-sensitive ordering of social interactions to understand how observed colony organization serves simulated resource flow through *C. pennsylvanicus* colonies. Integrating this suite of approaches shows that ant colonies are indeed segregated, though in a way more nuanced than previously theorised. Our work serves as a useful null model of a complex social insect society in the absence of perturbation.

## Methods

### Ant colony set-up and filming

Two queen-right *C. pennsylvanicus* colonies were collected from field sites in Centre County, central Pennsylvania, U.S.A. in December 2012. Seventy-five worker ants were selected from each colony and were individually labeled. Labels consisted of numbers printed on photo paper that were affixed to the ants’ posterior abdomens (gasters) with optically clear nail polish. Following a 5-minute acclimatization period, the labeling was not observed to alter the ants’ behaviours, movement or interactions.

The labeled ants and the queen were housed in a nest set-up consisting of a four-chambered wooden nest (total area = 63 cm^2^) that was gridded to a resolution of 1 cm^2^ and covered with a plexiglas top (video S1). The nest was contained within a filming box so that nest conditions were always dark. The nest was separated from a sand-bottomed foraging arena (total area = 144 cm^2^) by a 4-m long maze. The length of the maze was observed to create a clear separation between workers allocated to foraging versus internal colony tasks. Inside the foraging arena, ants had *ad libitum* access to water, 20% sucrose solution and a protein source (mealworms).

Each colony was filmed for approximately 30 minutes beginning at 21:00 hrs for 8 consecutive nights in June 2013 using a GoPro Hero2 camera with a modified IR filter (RageCams.com) illuminated under infrared light. Ants cannot detect infrared light, so the set-up was similar to the dark within-nest conditions that they naturally experience.

### Video analysis and trophallaxis measurements

For each night of filming, all trophallaxis interactions of each individual ant inside the nest were recorded for a 20-minute observation window. A trophallaxis event was recorded when ants engaged in mandible-to-mandible contact for greater than 1 s (video S2). A liquid food bubble transferring between the two individuals was usually observed accompanying this behaviour. While knowing the directionality of food exchange is important, it could not always be established through our observations and thus we do not analyze directionality. All together, the filming led to 401 hours of observation (76 ants × 2 colonies × 0.33 hours × 8 nights). The identities of the individuals interacting, the start and stop time of their trophallaxis interaction, and the grid location of their interaction within the nest was recorded. Additionally, the overall functional classification of every ant during each observation period was recorded (i.e. active forager, inactive forager, nest worker, queen- see below).

### Ant functional categorisation

Nest workers were ants that were never observed to leave the nest in the current or any previous observational periods. Active foragers were ants that actively entered or left the nest during the observation. Inactive foragers were ants that had been observed leaving the nest on previous nights, but which did not leave the nest during the current 20-minute period in which they were being analysed. Here ‘inactive’ simply refers to the fact that those ants were not actively outside the nest during the current observation period. It does not imply overall behavioural inactivity. The functional categorisation of an individual each night changed based on what they were doing in that observation period as well as what their behavioural history over previous observations had been (ie. once an ant has been observed foraging, it can no longer be classified as a ‘nest worker’).

### Trophallaxis count and duration

**Figure 1:**
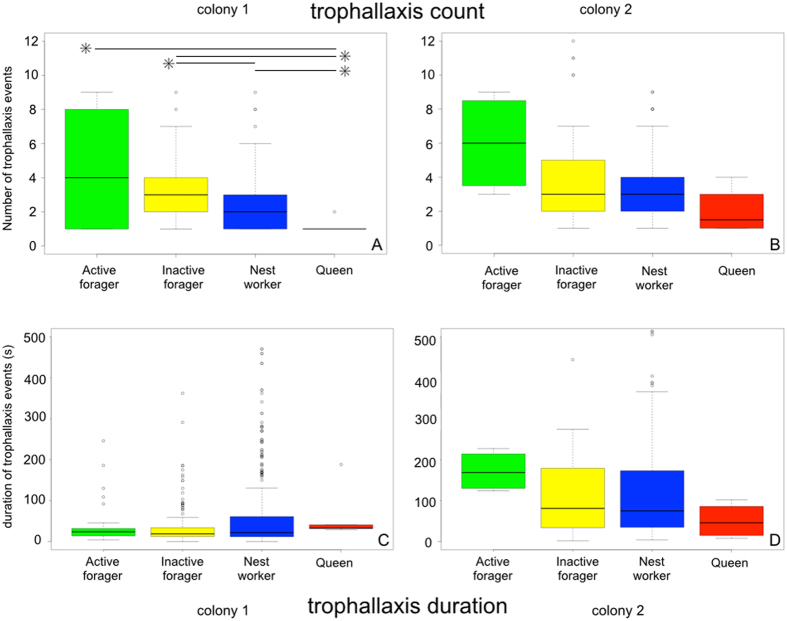
Trophallaxis count and duration. (**a,b**) Trophallaxis count and (**c,d**) duration as a function of ant behavioural classification. Asterisks represent statistically significant differences between groups (Table S3b). Black lines represent the median values, boxes represent the range of values in the 1^st^ through 3rd quartiles, and whiskers represent 1.5 times the inter-quartile range.

The number of trophallaxis events and their duration for each individual was recorded as above. To test for differences in mean trophallaxis count and duration as a function of ant functional classification (i.e. active forager, inactive forager, nest worker, or queen), two-sided Kruskal-Wallis one-way analysis of variance tests (hereafter, ‘K–W’) were conducted using the kruskal.test function in R^42^ (Fig. 1, Table S3a). For KW tests that were statistically significant (p < 0.05), Dunn’s tests coupled with a Benjamini-Hochberg correction for multiple hypothesis testing were then used to determine which functional classes had significant pair-wise differences (Table S3b). Data from all nights of observation were pooled together, but each colony was analysed separately.

### Static network analysis and visualization

Unweighted, undirected static network analyses were conducted using the iGraph package implemented in R^42^,^43^ such that only the number of discrete trophallaxis events and not their duration was used in the analysis. Network analyses were aimed at identifying whether key individuals that could serve as brokers or attenuators of food and/or disease flow were associated with a particular ant functional class; metrics analysed included degree, betweenness centrality, closeness centrality, and Burt’s constraint^44^,^45^. Degree is the number of connections that an individual ant has to other ants. Betweenness centrality is an estimate of how important an individual ant is to promoting connectivity across the entire colony^46^ and is measured by the number of times an individual acts as a bridge along the shortest path between two other ants. Closeness centrality is based on the distance (measured by shortest paths) from an individual to every other individual in the colony^44^: the more central an ant is, the lower its total distance is from all other ants. Burt’s constraint is a more nuanced measure to qualify; it measures the extent to which an ant’s interaction partners are redundant and thus identifies which ants could act as brokers of food or disease between ‘structural holes’ in a network^47^.

These metrics were analysed separately for each individual in each colony for each night of observation. Kruskal-Wallis tests were used to test whether there were differences in these metrics between ant functional groups because these metrics were not normally distributed (Table S4b). On metrics in which the K–W tests were statistically significant (p < 0.05), Dunn’s tests with p-values adjusted using a Benjamini-Hochberg correction were performed to assess which functional groups were significantly different from one another (Table S4c).

Trophallaxis networks were visualized using the circular layout in GEPHI^48^, in which circles represent individual ants (Fig. S1). Each ant was assigned a position based on its tracking ID that was maintained in all visualizations for all nights; trophallaxis interactions between ants are represented as lines (edges) between the respective circles. The length of an edge conveys no information, but the width of a given edge is proportional to the number of distinct trophallaxis interactions those ants had during that night of video observation.

### Functional group networks

**Figure 2:**
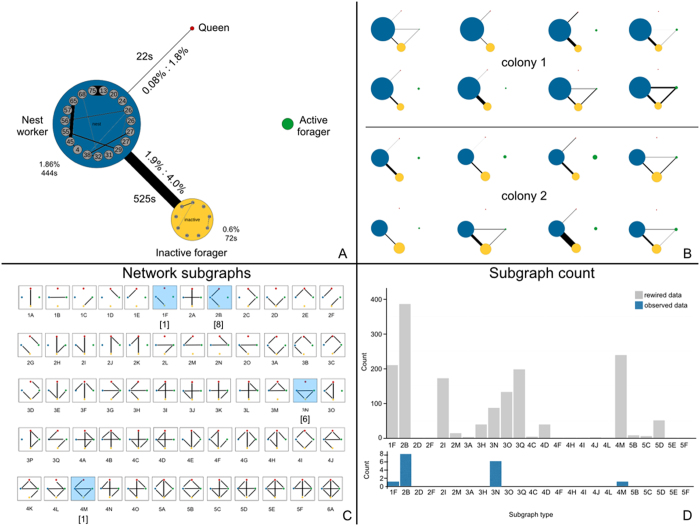
Ant function networks. (**a**) Representative ant functional group network showing trophallaxis interactions within and between ant functional groups. The total duration of all interactions between groups is given on the edge along with the percentage of the ant group’s total time budget that this duration represents. (**b**) All ant functional group networks for both colonies over all nights. (**c**) All possible functional group interaction patterns (network subgraphs); blue shading indicates which subgraphs were actually observed over the eight nights of observation for both colonies. (**d**) Histogram of expected count of subgraph types under network randomisation (grey) and the actual subgraph types realised in the study (blue).

To better understand the functional connectivity of the ant trophallaxis networks, we constructed *function networks* by collapsing each ant functional group (active foragers, inactive foragers, nest workers, queen) into nodes and representing the total duration of all interactions between each group as connections between nodes (weighted edges) (Fig. 2A,B). Here, the width of edges is proportional to the total duration of trophallaxis spent between those two functional groups and these are thus weighted network graphs.

### Ant-time calculation

While the total duration of trophallaxis (edge weight) in the ant functional group networks are the same for each group in the interacting pair, this time actually represents variable *per-capita* disease transmission risk due to different numbers of ants in each functional group and variable amounts of time in the nest for the active forager class. Thus, to get a more accurate understanding of how trophallaxis duration corresponds to potential per-capita transmission risk, we standardised these total trophallaxis durations using the epidemiological concept of ‘person-time’^41^, hereafter referred to as ‘ant-time’. For a detailed explanation of how ant-time was calculated, please refer to the SI.

### Proportion of time-budget engaged in trophallaxis

**Figure 3:**
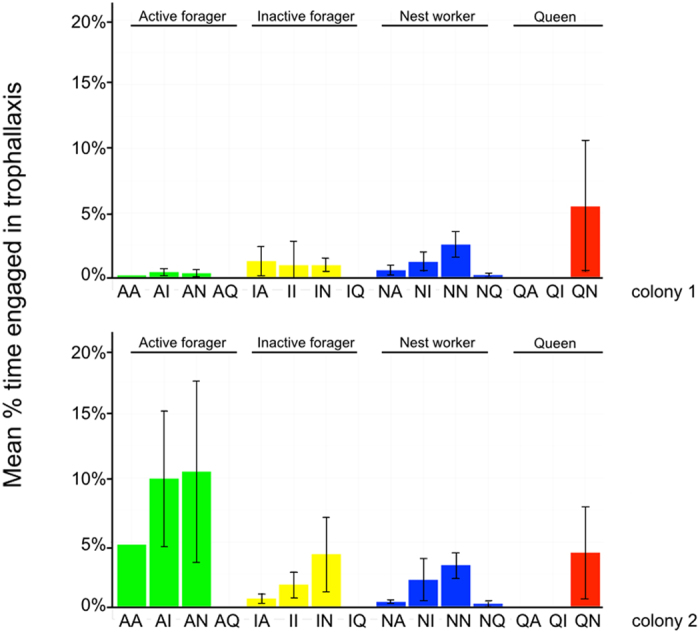
Trophallaxis as percentage of total ant-time budget. Barplots showing the mean percentage of total time budget for each dyadic interaction between ant functional groups over all eight nights for each colony, error bars are +/− st. dev.

Having calculated the ant-time for each class for each night, the percentage of each functional group’s total time-budget engaged in trophallaxis was calculated. The total duration of trophallaxis (edge weight) between two ant types provides the numerator and this is the same for both functional groups in the dyadic interaction being considered. However, the ant-time denominator varies for each type and thus the same total trophallaxis duration represents different total percentages of each group’s total time-budget available for interaction. The percentage time-budget for each dyadic interaction (for example, active forager - active forager, active forager - inactive forager, active forager -nest worker, active forager - queen) is presented in Fig. 3. For percentage of time-budget engaged in trophallaxis, refer to Table S5.

### Ant functional network subgraphs

Understanding the functional connectivity between active foragers, inactive foragers, nest workers and the queen is important for a broader understanding of how resource and disease flow is accomplished in carpenter ant societies. To facilitate comparison of these functional networks across nights and between colonies, we categorised the empirically observed network subgraphs. Network subgraphs are the different patterns of connection that can occur between nodes and provide an overview of global network structure^49^. The full range of possible network subgraphs for a 4-node network (i.e. active forager, inactive forager, nest worker, and queen) with varying numbers of connections (i.e. 1–6 edges) and the network subgraphs that were empirically observed are given in Fig. 2C.

To compare the observed network subgraphs to those expected under a null model (ie. trophallaxis connections within the colony are formed randomly), we generated simulated networks. Since there were both differing numbers of individuals in each functional category and differing numbers of interactions for each night, all network subgraphs are not expected to be equally realised. We generated randomised networks that preserve the same number of edges, degree distribution, and number of individuals in each ant functional class by rewiring the edges of each empirical network using double-edged swaps^50^ (implemented by iGraph’s ‘rewire’ function; self-loops were not permitted). For each night, we performed 500 random edge swaps to produce a new, randomised network realisation. We did this 100 times per observed night to generate a histogram of expected (N = 1,600) versus realised (N = 16) subgraphs, given in Fig. 2D.

### Spatial movement analysis

**Figure 4:**
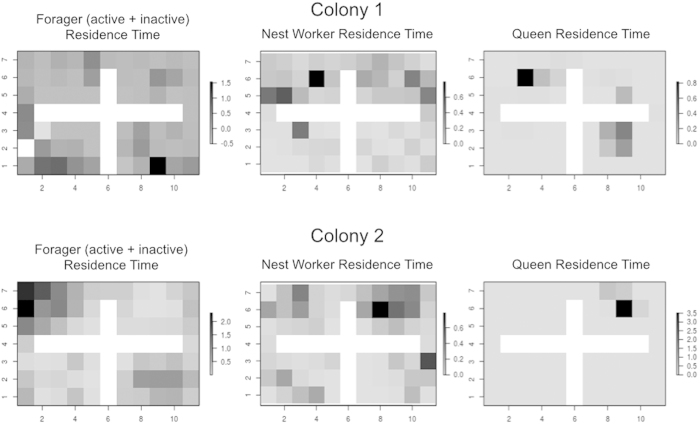
Segregated Use of Nest Space. Aggregated residence times in ant-days for queens, foragers (active and inactive), and nest workers for colonies 1 and 2.

Five known forager ants (here, active foragers and inactive foragers are grouped because individuals often transitioned between those categories over the eight nights of observation), five randomly selected nest workers, and the queen were chosen from each colony for additional spatial movement analysis. The wooden nest in which ants were housed was gridded to a resolution of 1 cm^2^, and the cell locations where the majority of the ant’s body was located as well as the time stamp when it was in that location were recorded for each observation period. The residence time spent in each cell was recorded to determine nest spatial use; this aggregated residence time is given in Fig. 4. This data was used to fit a continuous-time discrete space random walk model (see model description, SI) for ant movement behaviour^51^,^52^, with the goal of identifying the relative spatial movement of foragers (active and inactive) vs. nest workers and the movement behaviour of these groups (location and speed) around the queen.

### Simulated resource spread

**Figure 5:**
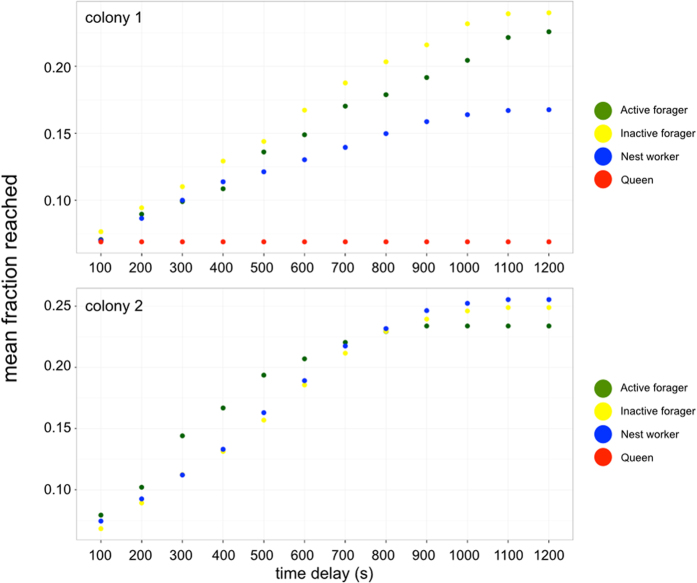
Simulated resource spread analysis. Mean fraction of the network reached over time when a theoretical food source is initiated from a given individual over the observed time ordered networks. Fractions are averaged by behavioural class over all 8 observation nights.

Interactions from the static networks were analysed with the additional inclusion of interaction time-stamps. Temporal networks were constructed using the package ‘timeordered’^53^ implemented in R (Figure S2). To understand how the pattern and timing of social interactions converge to impact the flow of food or disease, a resource-spread analysis was conducted using the *spreadanalysis* function in the ‘timeordered’ package. Using the empirical temporal networks, the *spreadanalysis* function randomly chooses an individual and ‘seeds’ it with a hypothetical resource at time 0. Then, using the time-ordered network interactions that were actually observed, the function simulates the fraction of the entire network reached by the theoretical food source through first-order, second-order, third-order etc. interactions at various time intervals. Here, we specified 100 s time intervals and the use of 20 randomly chosen individuals for each colony-night combination. The mean fraction of the network reached at each interval as a function of ant functional classification was computed for each colony averaged over all nights of observation and is given in Fig. 5.

**Data Accessibility.** Raw network data is available online and can be accessed through Dryad.

## Results

### Individual trophallaxis count and duration

There was a significant difference in the number of distinct trophallaxis events between ant functional groups in one colony (colony 1: χ^2^ = 20.34, p < 0.0002, two-sided K–W test, Fig. 1, Table S3a). Active foragers engaged in more trophallaxis events than the queen (z-statistic = −2.92, p < 0.004, Dunn’s test, Table S3b). In colony 1, inactive foragers engaged in more trophallaxis events than did either nest workers or the queen, (z-statistic = −3.420, −3.324 and p < 0.002, p < 0.002 nest workers and queen, respectively, Dunn’s test, Fig. 1, Table S3a) but there was no significant difference between active foragers and inactive foragers [z-statistic = −0.197, p = 0.4216, Dunn’s test, Table S3b]. In colony 2, trophallaxis count was not significantly different between ant functional groups (χ^2^ = 7.282, p = 0.063, two-sided Kruskal-Wallis test, Fig. 1, Table S3a). The duration of these trophallaxis events was not statistically different between ant functional groups in either colony (colony 1: χ^2^ = 6.4096, p = 0.0933, colony 2: χ^2^ = 4.386, p = 0.2227, two-sided K–W test, Table S3a).

### Static network analysis

Static, undirected networks for each colony for each night of observation are presented in Fig. S1. We tested for differences in network metrics aggregated over all nights between the different groups (active foragers, inactive foragers, nest workers, and queen). Active foragers and inactive foragers had a higher mean degree centrality (number of unique individuals interacted with, Table S4a) compared to nest workers in both colonies (Active foragers colony 1: z-statistic = −2.514, p < 0.02, colony 2: z-statistic = −2.4918, p < 0.02; inactive foragers colony 1: z-statistic = −4.432, p < 0.0001, colony 2: z-statistic = −3.836, p < 0.0005, Dunn’s test, Table S4c), but they are not significantly different from each other (colony 1: z-statistic = −0.4747, p = 0.3175, colony 2: z-statistic = −1.4411, p = 0.1122, Table S4c). While the queen had a median degree of 1, the identity of the individual she interacted with was not consistent across all nights in either colony. In colony 1, the closeness centrality of inactive foragers was significantly lower than that of nest workers (z-statistic = 3.391, p < 0.00001, Dunn’s test, Table S4c), which indicates that in that colony inactive foragers are more socially central on average than other colony members. There were no significant differences in closeness centrality between ant types in colony 2 (*χ*^*2*^ = 3.868, p = 0.2761, K–W test, Table S4b). The Burt’s constraint (redundancy of contacts) of the queen was significantly higher than that of active foragers and inactive foragers in colony 1 (z-statistic = 2.2875, 2.703, p = 0.0222, 0.0206, respectively, Dunn’s test, Table S4c), but not significantly different in colony 2 (*χ*^*2*^ = 3.7467, p = 0.2901, K–W test, Table S4b).

### Ant functional group networks

Of the 59 possible ant functional group network motifs, only 4 motifs were empirically observed in the 16 nights of observation across both colonies (Fig. 2C). Of these, 2 motifs in particular (motifs 2B, 3N) accounted for 87% of all empirically observed motifs. While the observed network motifs fall within those predicted to occur assuming random interactions between ant functional categories (Fig. 2D), whether these occur statistically more often than would be predicted cannot be tested as there is no variance available for the realisation of each night’s empirical network.

### Percent time-budget calculations

The percent time-budget each functional class engaged in trophallaxis with all other ant functional classes is given in Table S4. Taking the ant-time of each functional class into account provides a more nuanced view of ant group interactions for the individuals *within* those functional groups. While interactions with the queen represent a small fraction of the total trophallaxis happening inside the nest (1.66%+/−2.85% and 1.46%+/−2.2%, colony 1 and 2 respectively), this was found to represent approximately 4–5% of *her* entire time budget across both colonies (5.5% and 4.17%, colony 1 and 2 respectively, Table S4). This represents a greater percentage of time at risk than would be expected if just the duration of her trophallaxis events out of the whole colony were considered. In colony 2, active foragers spent an average of 25% of their time within the nest engaging in trophallaxis, compared to 5.82% for nest workers.

### Ant movement and spatial analysis

The average spatial usage of foragers (active and inactive), nest workers, and the queen is given in Fig. 4. Foragers (active and inactive) occupied a greater proportion of the total nest space than did either nest workers or the queen. The queen was largely immobile in both colonies, though in one colony (colony 1), the queen spent some time in three of the four chambers of the nest.

Results of our movement analysis show that in colony 2 nest workers are more mobile (have higher movement rates) than foraging ants while the latter are in the nest (p < 0.01, two-sided T-test, Table S5). This result does not hold in colony 1, and the overall effect size is small. There was no evidence of directional queen avoidance by foragers (active and inactive) or nest workers in either colony, but there was evidence in both colonies that foragers (active and inactive) move faster than nest workers when near the queen compared to when they are in another chamber (p < 0.01, two-sided T-test, Table S6).

### Simulated resource spread

Social network data has traditionally been analysed as a time-aggregated or static graph (Fig. S1) in which the timing and order of interactions is ignored. However, such timing and order is crucially important for dynamic flow processes, such as disease transfer^36^. Based on the timing of interactions, returning active foragers were never actually observed to interact in a way necessary for disease transmission to the queen (Fig. S2, i.e. after an active forager has returned from a trip outside the nest). Spread analysis indicates that such temporal segregation does not appear to inhibit the simulated flow of food, with no apparent differences between the mean network fractions reached when seeded by different ant types in either colony. Food generated from active foragers and inactive foragers in colony 1 was able to reach a higher mean fraction of the network than food generated from either nest workers or the queen (Fig. 5). In colony 2, food seeded from an active forager initially reached a higher mean fraction of the network, but this converged to ~25% by the end of the twenty minute observation period regardless of what functional class the food was seeded from.

## Discussion

Interacting with foragers, both socially and spatially, is a necessary risk for ant colonies. Theory predicts that interactions between potentially exposed foragers (actively returning foragers and inactive foragers that have foraged in the recent past) and other classes (i.e. nest workers) should be minimized^18^, but an integrated understanding of colony organization has remained elusive due to the inherent difficulties of observing within-colony social dynamics. Our behavioural analyses show that active foragers engage in more trophallaxis interactions than nest workers (approximately 2 additional interactions, Fig. 1a,b). Static social network analyses complement these findings; in addition to engaging in more trophallaxis events (higher degree), foragers (active and inactive) exchange food with a greater number of unique individuals (SI Table 2), indicating that their contact redundancy is lower than theory would predict^18^,^25^.

Re-analyzing the static network data though ant functional group networks (Fig. 2a,b), in which trophallaxis connections are represented between functional groups rather than individuals, allows broader patterns of colony social organization to emerge^8^. Importantly, it also facilitates network comparison across nights and colonies and likely reflects a scale of analysis more biologically relevant to whole colony functioning^8^. Two network subgraphs predominate, accounting for 87% of those observed: inactive-nest-queen and forager-inactive-nest closed triad (Fig. 2c,d); these represent only two out of 59 possible patterns of functional group connection. From these subgraphs, it appears that inactive foragers may play an important role as brokers of trophallaxis in carpenter ant colonies, and that active foragers are interacting with nest workers more than might be expected.This becomes even clearer when ant-time standardization is taken into account. When the higher abundance of nest workers is taken into account, active and inactive foragers spend a greater per-capita percentage of their total in-nest time budget actually engaged in trophallaxis (Table S4). While this is an intuitive finding should they only be spreading beneficial resources, this is less intuitive given the variety of material that can be spread through intimate social contact (Table S1) in social insect colonies. Thus, this finding sheds light on the balance of constraints that have shaped the structure of carpenter ant social structure over evolutionary time. This suggests that under conditions in which perturbation is not present through disease or resource competition, organizational immunity is not accomplished through simply reducing trophallaxis with potentially exposed members. Future studies in which the balance is empirically ‘tipped’ in favor of disease transmission will shed light on how malleable this social structure is in the face of acute perturbation.

In addition to the social position of foragers within the colony, we were also interested in how they spatially occupied their nest environment. Analysis of nest spatial usage showed that foragers (active and inactive) use more nest area relative to both nest workers and the queen (Fig. 4). While the queen’s lack of movement synchronizes well with predictions from social immunity (i.e. to be secluded) the expansive movement of the foragers is counterintuitive. It is reasonable to assume that foragers should avoid internal areas of the nest^18^,^20^ but we did not observe this (Fig. 4). However, we found evidence in both colonies that foragers could be modulating their speed in response to their social environment (Table S4). When foraging ants were in the same chamber as the queen, they moved faster than their nest worker counterparts. Such speed modulation could potentially reduce the impact of pathogen transmission to which foragers may have been exposed by moving faster near the most important individuals (i.e. queen, younger workers). This has not been previously considered as a mechanism to mitigate disease spread within the nest. Future studies that specifically address whether different ant classes alter their speed in response to different social environments inside the nest and what, if any, biological impact this has for potential disease transmission are needed.

The static network analyses of colony social organization and the spatial movement of foragers reveal that active and inactive foragers engage in more trophallaxis interactions and occupy a great spatial area within the nest than their nest worker and queen counterparts. This would appear counter to the verbal models of social immunity where intuition, without within-nest behavioural data, has suggested a stronger segregation between worker types, especially foragers^18^. In considering social immunity, however, time has been a previously neglected component^20^. In our data, when the timing and order of trophallaxis interactions are taken into account, active and inactive foragers and the queen never interact in a way that could lead to the biologically meaningful transfer of disease (i.e. after a forager has come back into the nest after a foraging trip, carrying some pathogen that might transfer to the queen via either oral food exchange or prolonged physical contact). Thus, the timing of social interactions provides additional evidence for nuanced organization within *C. pennsylvanicus* colonies.

Infectious agents are not the only pressure that ant colonies face. Since trophallaxis interactions are a conduit for disease and food (and other material, see Introduction), there is a fundamental tradeoff in optimizing food flow while minimizing disease spread, both of which are brought into the colony by foragers^13^. This tradeoff is of interest because it could impact colony functioning even in the absence of disease; the temporal segregation of foragers could prolong the time it takes for incoming food to reach other colony members. Simulations of resource spread over the empirically observed trophallaxis networks (Fig. 5) provide an understanding of the flow tradeoff that could result from temporal segregation of foraging ants. In both colonies the mean fraction of the network reached a high of 20–25% after 20 minutes. In colony 1, this was achieved in food originating from active and inactive foragers, showing that their temporal segregation did not appear to impact the potential flow of food. In colony 2, food saturated at this percentage regardless of what ant type it originated from.

Our study also highlights the need to consider an ant’s behavioural past when considering its present and future role in colony functioning and the introduction of disease risk. Active foragers who have just returned from outside the nest are clearly capable of introducing potential pathogens into the colony (Table S1). What remains unclear is for how long they remain capable of transmitting disease- at what point does a potentially infected incoming forager transition to an inactive forager no longer posing a threat? While the classification of inactive forager used here was defined by the methods of the study, there are clear behavioural differences between these ants and nest workers who have never been observed to leave the nest. Had they been included within the nest worker categorisation, this would have obscured the network and spatial signals observed. We advocate that future studies of disease transmission in social insect societies follow individuals over a time period that will capture past exposure, and thus the continuum of foraging behaviour and pathogen risk.

## Conclusion

Through the incorporation of dyadic- and network-level social interactions, individual movement data, and the timing of social interactions, we have gained insights into how colony organization is accomplished in *C. pennsylvanicus* colonies. The standing organization in an ant society, exemplified by the carpenter ant colonies studied here, appears to be more nuanced than previously imagined. Measuring social and spatial segregation in tandem is important because pathogens may have alternate means of gaining entry to, and transmitting within, social insect societies (Table S1) and it remains unclear to what extent spatial distance is a proximate mechanism underlying social distance. The timing of social interactions may provide an additional layer of disease prophylaxis in social insect societies. This adds evidence for the growing argument that temporal information and meaningful behavioural interactions should be included into social network analyses if we are to make biologically accurate conclusions^27^,^54^. The temporal component of social interactions is especially worth future investigation because it is unclear to what extent pathogen infectivity and/or infective dose is reduced over time or multiple trophallaxis passages. Thus, social insect societies which employ living food ‘silos’^39^ may indeed be making use of such temporal protection.

Analyzing carpenter ant colony organization and functioning in the absence of disease and environmental heterogeneity provides a useful null model; we are now primed to study how these complex systems react to perturbation. Future experiments in which laboratory infections are combined with integrated social, spatial, and temporal approaches will further inform how social insect colony organization and individual behaviour dynamically interact to reduce disease transmission. Social insects are host to a range of both generalist and specialist parasites^13^, some of which can change the behaviour of the infected host (ie. *Ophiocordyceps*) or potentially alter the interactions between healthy and infected nest mates (ie. *Beauveria, Metarhizium*), and some of which cause no discernible change in behaviour. Such studies will also afford us the ability to synchronize theoretical predictions about disease transmission in societies from agent-based and SIR modeling approaches^55^,^56^ with empirical data from experiments in which disease can actually be introduced.

## Additional Information

**How to cite this article**: Quevillon, L. E. *et al.* Social, spatial, and temporal organization in a complex insect society. *Sci. Rep.* **5**, 13393; doi: 10.1038/srep13393 (2015).

## Supplementary Material

Supplementary Information

Supplementary Video S1

Supplementary Video S2
