# Supplementary material for: Social, spatial, and temporal organization in a complex insect society
Source: Sci Rep. 2015 Aug 24;5:13393. doi: 10.1038/srep13393 (PMC4547134; doi:10.1038/srep13393)
Supplement: Supplementary Information [file srep13393-s1.pdf]

Supplementary Information for:

Social, spatial, and temporal organization in a complex insect society.

Lauren E. Quevillon[1,2*], Ephraim M. Hanks[1,3], Shweta Bansal[4,5] and David P. Hughes[1,2,6*]

1 Center for Infectious Disease Dynamics, Penn State University, University Park, Pennsylvania, USA, 2 Department of Biology, Penn State University, University Park, Pennsylvania, USA, 3 Department of Statistics, Penn State University, University Park, Pennsylvania, USA, 4 Department of Biology, Georgetown University, Washington, D.C., USA 5 Fogarty International Center, National Institutes of Health, Bethesda, MD, USA 6 Department of Entomology, Penn State University, University Park, Pennsylvania, USA

*** Email: leq103@psu.edu, dph14@psu.edu**

Supplemental text

Ant-time calculation:

To calculate ant-time, we took the number of ants in each functional group for each night of observation and multiplied by the total time they were in the nest and therefore available to engage in trophollaxis interactions with other ants. The queen, nest workers, and inactive foragers were by definition in the nest for the entire 20-minute observation period each night and accordingly the calculation of ant-time is a simple product of the number of those ants by the 1,210-second observation window. However, foragers were in the nest for variable amounts of time and so ant-time for the forager class is calculated by summing how much time each individual forager was in the nest for a precise calculation of the time they were available for within-nest interactions. The ant-time formulas are given below:

Foragers: $T_F = \sum_{F_i}^{F_n} t_i$, where t_i is the amount of time *forager* F_i spent inside the nest.

All others: $T_{ant} = N_{ant} * 1210\text{s}$, where N_{ant} = number of ants in type *ant*.

Ant movement model:

In both colonies, the observed residence times in each grid cell and transitions to neighboring cells were used to fit a continuous-time discrete-space random walk model for ant movement behaviour and used to calculate a movement or transition rate between cells. We used a continuous-time discrete-space (CTDS) agent-based random walk model38, 42 to make inference about ant movement behaviour. The CTDS framework is notable in that it allows for inference on both directional (e.g., queen avoidance) and location-based (e.g., variable movement rates in different nest chambers) movement mechanisms. Drawing on standard continuous-time Markov chain models (e.g., 42), if an ant is in cell i at time t, then define the rate of transition from cell i to a neighboring cell j as λ(ij). The total rate λ(i) at which ants move (transition) out of cell i is the sum of the rates to all neighboring cells: $\lambda i = \lambda ij$, and when the ant moves, the probability of moving to cell k (instead of to another neighboring cell) is the ratio: λik / λ*i*.

To model ant movement behaviour near the queen, we will model λ(ij) as a function of a spatial covariate that measures the distance from the queen's most used locations ('Distance From Queen'- DFQ) at each grid cell. To examine local behaviour, the DFQ covariate was set to be constant out of the queen's chamber. The DFQ covariate is location-based and will allow us to model differences in movement rates when near or far from the queen. We also considered a directional covariate, a gradient of the DFQ covariate (GDFQ). The GDFQ gradient is a directional vector that points towards the queen, or along the direction of steepest ascent of the DFQ covariate, and the GDFQ covariate will be different for the transition rates to neighboring cells in different directions, thus allowing for directional preference in ant movement. We also consider potential differences in movement behaviour between foraging (F) and non-foraging (NF) ants, with F=1 for foraging ants and F=0 otherwise, and NF=0 for foraging ants and NF=0 otherwise. We model the movement rate λk(ij) of the k-th ant from cell i to cell j as a function of interactions of these covariates and corresponding regression parameters {β}:

$\lambda_k(ij) = \exp\{ F_k\beta_1 + NF_k\beta_2 + (F_k * DFQi)\beta_3 + (NF_k * DFQ_i)\beta_4$
$+ (F_k * GDFQ_{ij})\beta_5 + (Fk * GDFQ_{ij})\beta_5 \}$

Differences in overall movement rates between foragers and non-foragers will be represented by differences in β_1 and β_2 , with positive values corresponding to higher movement rates. Positive values of β_3 correspond to higher movement rates of foraging ants when far from the queen, and decreased movement rates near the queen. Positive values of β_5 correspond to preferential directional movement by foragers away from the queen (in the direction of the increase in

the gradient of DFQ). The parameters β_4 and β_6 correspond to the response of non-foraging ants to DFQ and GDFQ, respectively. Hanks et al. (2013) have shown that inference on the parameters in this movement model can be accomplished using a Poisson GLM, which we fit using the 'glm' command in R.

Data availability:

Raw network data for colony 1 and colony 2 over all 8 nights of observation is available online at Dryad.

Supplemental Tables and Figures

Video S1: Ant nest set-up.

Video S2: Trophallaxis montage.

Table S1: Major parasites of ants
A non-exhaustive list of the major parasite taxa infecting and/or transmitting within ant colonies. The mechanism of entry into the colony (if known) is given as well as the major route of transmission once the parasite is inside the colony (if known). The ant life-history stage predominantly infected is also given.

Table S2: Selected summaries of recent social insect research

Table S3: Trophallaxis count and duration statistics
(a) Two-sided Kruskal-Wallis tests and (b) Dunn's tests differences in trophallaxis count and duration as a function of ant functional classification. Asterisks represent statistically significant differences between groups following a Benjamini-Hochberg correction for multiplicity of hypothesis testing.

Figure S1: Static trophallaxis networks.
Unweighted, bi-directional trophallaxis networks for all 8 nights for a) colony 1 and b) colony 2. Individual ants are represented as circles; their x-y coordinates were randomly generated and maintained in all graphs. Lines between circles represent a trophallaxis interaction between those ants; the length and width of the line conveys no additional information.

Figure S2: Time-ordered trophallaxis networks.
Unweighted, bidirectional, time-ordered networks for all 8 nights for a) colony 1 and b) colony 2. Individual ants are represented as vertical lines moving through time (time starts at y=0 and moves forward in the +y direction). Horizontal lines represent the start time of trophallaxis interaction between the two individuals connected. Active foragers are shaded in green, inactive foragers are shaded in yellow, nest workers are not shaded, and the queen is shaded in red.

Table S4: Network metrics by ant behavioural class.
a) Mean static network metrics (degree, betweeness, closeness, and constraint) for each ant functional group b) Kruskal-Wallis test results and c) Dunn's test statistics for static network metrics as a function of ant type. Network metrics were not weighted by the duration of trophallaxis, only trophallaxis count.

Table S5: Percentage of time budget engaged in trophallaxis.
Mean and standard deviation of percentage time budget engaged in trophallaxis for each functional group comparison. The functional group on the left-most side in each label is the focal group, and it is their ant-time used in the denominator.

Table S6: Inference on ant movement parameters.
Inference from a continuous-time random walk model of ant movement in two ant colonies (See Fig. 4)

Table S1: Major parasites of ant colonies

Adapted from Schmid-Hempel 1998, Tables 3.1, 3.2 and Appendix 2 and references therein

Parasite			Entry	Within-nest transmission mode	stage infected
**Trematoda**					
	Dicrocelidae		per os	per os	brood
**Nematoda**					
	Mermithidae		direct host penetration; per os	direct host penetration; per os	brood
	Rhabditida		carried into nest, per os		workers
**Cestoda**					
	Anomotaenia brevis			per os	brood
**Protozoa**					
Gregarines			per os		
**Bacteria**					
	Pseudomonas				
**Fungi**					
	Ophiocordycepitae		contact	none	workers
	Clavicipitaceae				
		Beauveria bassiana	contact, per os	contact, per os	workers, brood
		Metarhizium anisopliae	contact, per os	contact, per os	workers, brood
	Entomopthoraceae				
		Entomopthora	contact		
		Pandora spp.			
	Trichocomaceae				
**Parasitic Insects**					
Hymenoptera					
	Ichneumonidae				
	Braconidae		oviposition near nest	burrowing into host	
	Eucharytidae		carried into nest	burrowing into host, ectoparasitic	larvae
Strepsiptera					
	Myrmecolacidae		carried into nest	burrowing into host	brood
Diptera					
	Phoridae		oviposition into worker	none	
	Tachinidae				

Table S2: Selected summaries of recent social insect literature

Reference	Year	Summary
Naug, D. & Smith, B. Experimentally induced change in infectious period affects transmission dynamics in a social group. *Proc Roy Soc B* **274**, 61-65 (2007).	2007	Naug and Smith 2007 fed known forager bees on sucrose solutions containing microbeads. They followed the first- and second-order trophallaxis interactions of these foragers and then dissected a random subset of the colony after a pre-determined exposure time to follow microbead transmission.
Otterstatter, M. C. & Thomson, J. D. Contact networks and transmission of an intestinal pathogen in bumble bee (Bombus impatiens) colonies. *Oecologia* **154**, 411-421 (2007).	2007	Otterstatter and Thompson 2007 followed the transmission of a pathogen through the physical contact network of bumble bee colonies. They investigated transmission through both naturally-infected foundresses (vertical transmission) and through infection initiated with infected foragers (horizontal transmission). The major drawback to this study is the small size of the colonies used; numbering between 4-6 workers and a queen. While this may represent a good approximation for disease transmission in founding colonies, it is likely not directly translatable to disease transmission dynamics within larger, established colonies.
Naug, D. Structure of the social network and its influence on transmission dynamics in a honeybee colony. *Behav Ecol Sociobiol* **62**, 1719-1725 (2008)	2008	Naug 2008 followed the first- and second-order trophallaxis interactions of known forager bees in a single colony. However, while the duration of these trophallaxis events was known, the actual amount of sucrose transferred was not directly measured and duration was used as a proxy.
Buffin, A., Denis, D., Van Simaeys, G., Goldman, S. & Deneubourg, J.-L. Feeding and stocking up: radio-labelled food reveals exchange patterns in ants. *PLoS One* **4**, e5919 (2009).	2009	Buffin et al. 2009 investigated food flow dynamics in ants at the level of the entire colony. Using radioactively labeled food, they followed the rate at which the radiographic signal spread through the colony over time, and where that signal spatially accumulates. While this study provides a great overview at the level of the colony, it remains unclear how this food flow is accomplished through dyadic level social interactions, and the social composition and relative timing of such dyadic interactions.
Sendova-Franks, A. B. *et al.* Emergency networking: famine relief in ant colonies. *Anim Behav* **79**, 473-485 (2010).	2010	Sendova-Franks et al. 2010 examined trophallaxis networks in colonies that were maintained in fed followed by semi-starved conditions. They distinguished between internal and external nest workers, and found that increased food flow after semi-starvation was mediated by the spatial movement of internal workers away from brood and movement of foragers deeper into the nest. They also found that under semi-starved conditions, internal workers transitioned from being primarily food receivers to both food receivers and donors, which they suggest could act as a mechanism to dilute potential poisons. Our work follows on this by employing time-ordered network analyses to investigate food flow.
Pinter-Wollman, N., Wollman, R., Guetz, A., Holmes, S. & Gordon, D. M. The effect of individual variation on the structure and function of interaction networks in harvester ants. *J Roy Soc Interface*, rsif20110059 (2011).	2011	Pinter-Wollman et al. 2011 looked at information exchange through spatial proximity of ants in the entrance chambers of colonies. From this they found that most ants only had a few interactions and very few ants had the majority of interactions. They also found that the majority of interactions were not randomly distributed but rather occurred in specific hotspots near the chamber entrance. Though this work does a fantastic job of investigating individual differences in spatial interactions, we don't know to what extent this variation also applies to social interactions and to other areas of the nest besides the entrance chamber.
Waters MJ S. & Fewell, J. H. Information processing in social insect networks. *PLoS One* **7**, e40337 (2012).	2012	Waters and Fewell 2012 created antennation networks for harvester ant colonies and compared the overarching network structure to those of networks from other systems (ie. technology networks, gene regulatory networks, etc.). They suggest that the network subgraph motifs they observe may have been selected for as a way to increase information flow. We employ similar motif analyses to look for evidence of social segregation in our present study.
Jeanson, R. Long-term dynamics in proximity networks in ants. *Anim Behav* **83**, 915-923 (2012).	2012	Jeanson, R. 2012 used RFID tags to follow the spatial movement of individual ants over the course of multiple weeks. From this, they built association networks based upon which ants were near each other. Importantly, this work showed that these spatial proximity networks were stable over time and robust to the removal of the queen. However, whether nest spatial usage correlates to actual social interactions between proximate individuals was not explicitly tested.

Table S3: Trophallaxis count and duration.

Table S3a: Kruskal-Wallis test on trophallaxis count and duration

	Colony 1		Colony 2	
	Chi-sq statistic	p-value	*Chi-sq statistic*	p-value
Count	20.3349	0.0001447	7.282	0.06343
Duration	6.4096	0.0933	4.386	0.2227

Table S3b: Dunn Test for differences in trophallaxis count between ant types

Comparison	Colony 1		Colony 2	
	z-statistic	p-value	z-statistic	p-value
Act. forager - In. forager	-0.197696	0.4216	n.s.	n.s.
Act. forager - nest worker	-1.802277	0.0429	n.s.	n.s.
Act. forager - queen	-2.920887	0.0035*	n.s.	n.s.
In. forager - nest worker	-3.420299	0.0019*	n.s.	n.s.
In. forager - queen	-3.324025	0.0013*	n.s.	n.s.
Nest worker - queen	-2.233482	0.0191*	n.s.	n.s.

*Asterisks indicate statistically significant p-values following a Benjamini-Hochberg correction

Figure S1

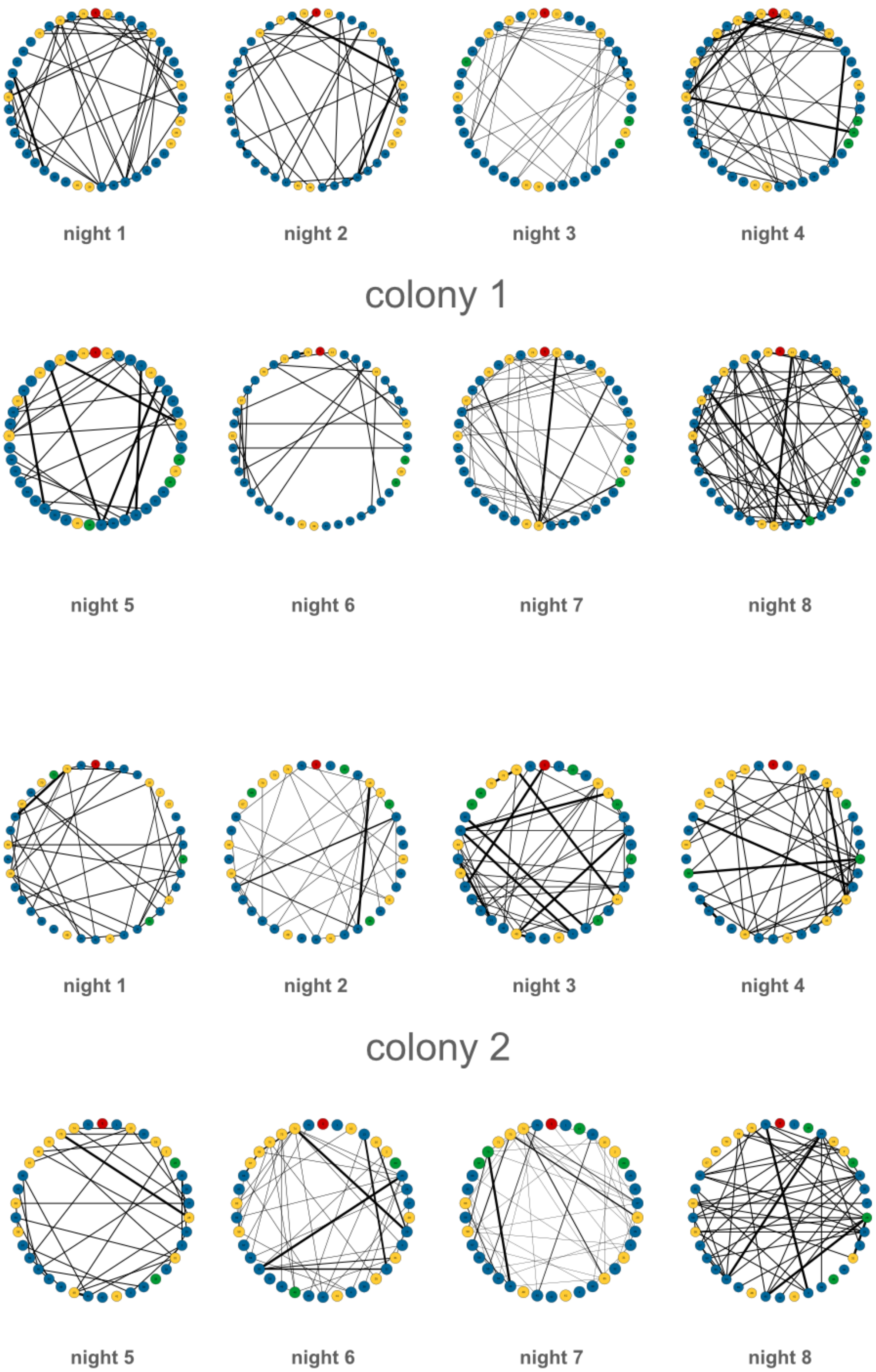
night 1
night 2
night 3
night 4
colony 1
night 5
night 6
night 7
night 8
night 1
night 2
night 3
night 4
colony 2
night 5
night 6
night 7
night 8

Figure S2

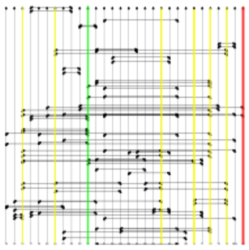
night 1

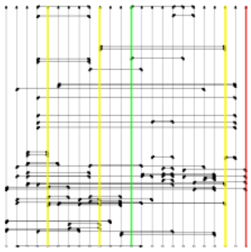
night 2

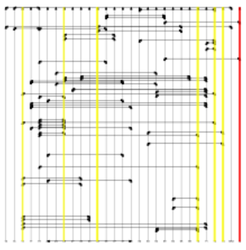
night 3

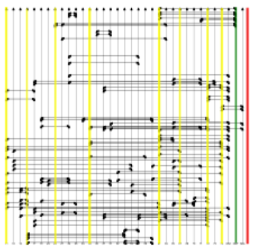
night 4

colony 1

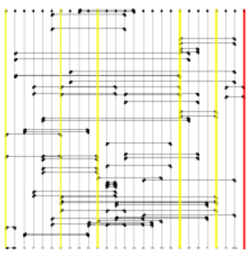
night 5

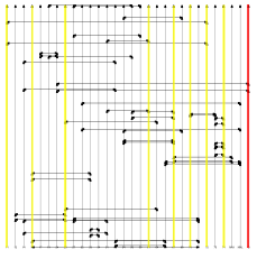
night 6

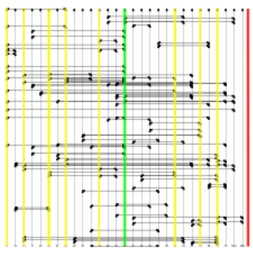
night 7

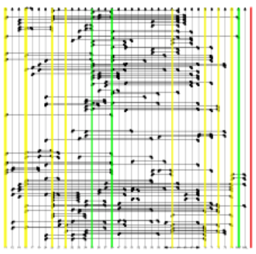
night 8

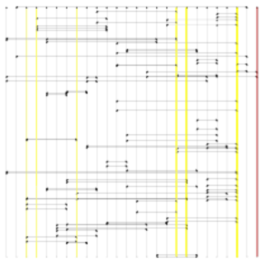
night 1

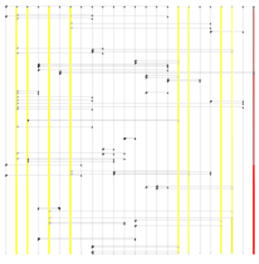
night 2

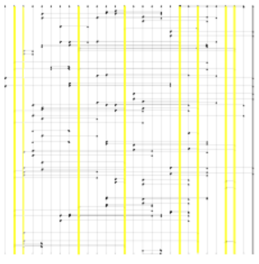
night 3

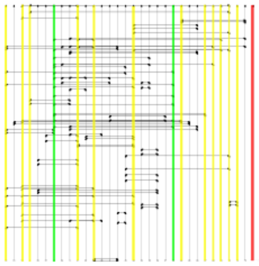
night 4

colony 2

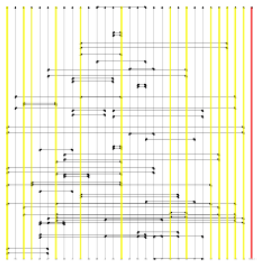
night 5

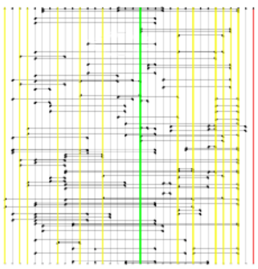
night 6

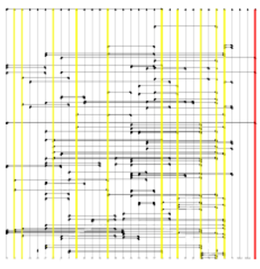
night 7

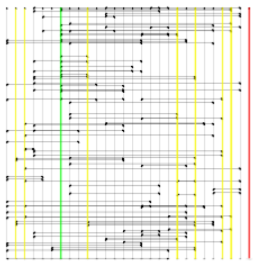
night 8

Table S4: Network metrics by ant behavioural class.

**Table S4a: Mean network metric values by ant functional class.**				
	**Colony 1**			
	**Active F.**	**Inactive F.**	**Nest W.**	**Queen**
Degree centrality	5.444	4.212	3.301	1.800
Betweenness centrality	34.773	30.509	21.611	8.400
Closeness centrality	0.008	0.006	0.009	0.003
Burt's constraint	0.553	0.508	0.604	0.900
	**Colony 2**			
Degree centrality	6.000	3.636	2.454	2.000
Betweenness centrality	61.035	29.826	23.963	15.018
Closeness centrality	0.009	0.010	0.008	0.011
Burt's constraint	0.361	0.547	0.540	0.687

**Table S4b: Kruskal-Wallis test on network metrics**				
	**Colony 1**		**Colony 2**	
	Chi-sq statistic	p-value	*Chi-sq statistic*	p-value
Degree centrality	25.0686	1.49E-05	19.9183	0.0001765
Betweenness centrality	14.9998	0.001817	8.3246	0.03976
Closeness centrality	15.164	0.001682	3.868	0.2761
Burt's constraint	11.1124	0.01113	3.7467	0.2901

**Table S4c: Dunn's test for differences in network metrics by ant type**				
	**Colony 1**		**Colony 2**	
	z-statistic	p-value	z-statistic	p-value
	**Degree centrality**			
Active F. - Inactive F.	-0.474675	0.3175	-1.441133	0.1122
Active F. - Nest W.	-2.514156	0.0179*	-2.491783	0.0191*
Active F. - Queen	-2.005933	0.0336	-1.980356	0.0477
Inactive F. - Nest W.	-4.432227	0.0000*	-3.836072	0.0004*
Inactive F. - Queen	-2.023584	0.043	-1.289485	0.1183
Nest W. - Queen	-0.583159	0.3359	-0.279067	0.3901
	**Betweenness centrality**			
Active F. - Inactive F.	-0.070616	0.4719	-1.17404	0.1442
Active F. - Nest W.	-1.612799	0.0801	-1.77347	0.0762
Active F. - Queen	-1.849027	0.0645	-1.852814	0.0959
Inactive F. - Nest W.	3.390584	0.0021*	-2.169286	0.0902
Inactive F. - Queen	-2.148224	0.0475	-1.380717	0.1255
Nest W. - Queen	-1.067462	0.1715	-0.818929	0.2064
	**Closeness centrality**			
Active F. - Inactive F.	-1.461944	0.0863	n.s.	n.s.
Active F. - Nest W.	-2.788372	0.0159*	n.s.	n.s.
Active F. - Queen	-2.381952	0.0172*	n.s.	n.s.
Inactive F. - Nest W.	-2.726254	0.0096*	n.s.	n.s.
Inactive F. - Queen	-1.710273	0.0654	n.s.	n.s.
Nest W. - Queen	-0.840669	0.2003	n.s.	n.s.
	**Burt's Constraint**			
Active F. - Inactive F.	0.028268	0.4887	n.s.	n.s.
Active F. - Nest W.	1.118676	0.158	n.s.	n.s.
Active F. - Queen	2.287563	0.0222*	n.s.	n.s.
Inactive F. - Nest W.	2.355095	0.0278*	n.s.	n.s.
Inactive F. - Queen	2.703288	0.0206*	n.s.	n.s.
Nest W. - Queen	1.969885	0.0366	n.s.	n.s.

*Asterisks indicate statistically significant p-values following a Benjamini-Hochberg correction

Table S5: Percentage of total time budget engaged in trophallaxis.

Comparison	Colony 1		Colony 2	
	mean (%)	sd	mean (%)	sd
Active F. – Active F.	0.131	NA	4.797	NA
Active F. – Inactive F.	0.373	0.283	9.956	5.295
Active F. – Nest W.	0.301	0.296	10.500	7.067
Active F. - Queen	NA	NA	NA	NA
Inactive F. – Inactive F.	0.914	1.829	1.702	1.014
Inactive F. – Active F.	1.237	1.168	0.616	0.385
Inactive F. – Nest W.	0.912	0.516	4.054	2.924
Inactive F. - Queen	NA	NA	NA	NA
Nest W. – Nest W.	2.534	0.988	3.197	0.992
Nest W. – Active F	0.529	0.408	0.350	0.113
Nest W. – Inactive F.	1.171	0.698	2.077	1.650
Nest W. - Queen	0.138	0.134	0.199	0.174
Queen – Active F.	NA	NA	NA	NA
Queen – Inactive F.	NA	NA	NA	NA
Queen – Nest W.	5.500	5.091	4.173	3.587

Table S6: Inference on ant movement parameters in a continuous-time random walk model of ant movement in two ant colonies (See Fig. 4)

	Effect	Estimate	Std. Error	p-value (T-test)
**colony 1**	F	-3.18845	0.04111	< 10^-15
	NF	-1.63299	0.03379	< 10^-15
	F*DFQ	-0.04696	0.04114	0.25371
	NF*DFQ	0.12605	0.03304	0.00014
	F*GDFQ	-0.02391	0.0412	0.5616
	NF*GDFQ	-0.0077	0.03221	0.81119
**colony 2**	F	-2.2599	0.02222	< 10^-15
	NF	-0.92576	0.02035	< 10^-15
	F*DFQ	-0.19091	0.02453	< 10^-14
	NF*DFQ	0.04115	0.01435	0.00416
	F*GDFQ	-0.01581	0.03571	0.65801
	NF*GDFQ	-0.00106	0.01023	0.91763
